# Emodin is a novel phosphatidylethanolamine anabolism inhibitor that reprograms lipid metabolism to overcome 5-fluorouracil resistance in colorectal cancer

**DOI:** 10.1016/j.jpha.2025.101343

**Published:** 2025-05-12

**Authors:** Yanyan Chen, Yanchen Liu, Zhicheng Gong, Zhaohui Huang

**Affiliations:** a Wuxi Cancer Institute, Affiliated Hospital of Jiangnan University, Wuxi, Jiangsu, 214062, China; b Laboratory of Cancer Epigenetics, Wuxi School of Medicine, Jiangnan University, Wuxi, Jiangsu, 214122, China

## Abstract

• Emodin overcomes 5-Fu resistance in colorectal cancer.
• 5-Fu drives PE accumulation to shift PEBP1 binding partners and activate MAPK signaling, which maintains drug resistance.
• Emodin acts as a novel PE anabolism inhibitor by directly targeting PIM1.
• Emodin reverses 5-Fu-induced PE accumulation to restore the PEBP1-RAF1 interaction to inactivate MAPK signaling.

## Graphical abstract

**Figure:**
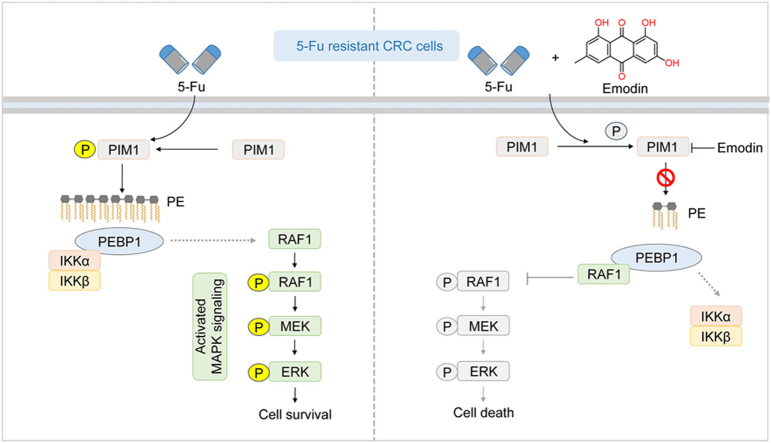

Colorectal cancer (CRC) is a pervasive health concern worldwide, ranking third in malignancy prevalence and second in cancer-related mortality. The primary chemotherapeutic agent for CRC is 5-fluorouracil (5-Fu). Unfortunately, drug resistance is almost inevitable in patients, significantly reducing its efficacy. Therefore, underlying the mechanisms of 5-Fu resistance and exploring reversal strategies are crucial. Rewiring of lipid metabolism is critical for cancer cell survival under drug threat [1]. However, the role of chemotherapy in inducing lipid metabolism reprogramming in 5-Fu-resistant CRC cells and its impact on cell fate remain unknown. Our study revealed that exposure to 5-Fu elicited lipid metabolism reprogramming in 5-Fu-resistant CRC cells, specifically leading to the accumulation of phosphatidylethanolamines (PEs). These accumulated PEs shifted the binding affinities of PE binding protein 1 (PEBP1) from Raf-1 proto-oncogene, serine/threonine kinase (RAF1) to inhibitor of nuclear factor kappaB (NF-κB) kinase subunits alpha/beta (IKKα/β), thereby activating the mitogen-activated protein kinase (MAPK) signaling pathway and maintaining cell survival upon acute 5-Fu treatment. Given that emodin, as an active phytochemical, has demonstrated various anticancer functions and can inhibit lipid anabolism in a high-fat-diet-induced obese mouse model [2], we speculated its potential to reverse 5-Fu resistance by modulating the lipid metabolism. Our data further revealed that emodin effectively reversed the 5-Fu-resistant phenotype *in vitro* and *in vivo.* Mechanistically, emodin inhibited 5-Fu-induced PE anabolism and reversed drug resistance by directly targeting Pim-1 proto-oncogene, serine/threonine kinase (PIM1) activation.

We first profiled the lipid metabolism signature in parental and 5-Fu-resistant cells upon 5-Fu treatment. 5-Fu-resistant CRC cell lines were constructed (Fig. S1), and a drastically altered lipid metabolic signature was observed in HCT-8^5-FuR^ cells but not in HCT-8 cells (Fig. S2A). The levels of most lipid metabolites in HCT-8^5-FuR^ cells were higher than those in HCT-8 cells, indicating that 5-Fu-resistant cells may accelerate their lipid metabolic rates to maintain survival under 5-Fu stress (Fig. S2B). Notably, the concentrations of various PEs increased following 5-Fu treatment in HCT-8^5-FuR^ cells (Figs. S2C and D). To gain further insights into how PE accumulation facilitates cell survival, we examined the transcriptome signatures in HCT-8^5-FuR^ and HCT-8 cells treated with 5-Fu. A total of 2,743 upregulated and 3,148 downregulated genes were identified in HCT-8 cells, whereas 201 upregulated and 292 downregulated genes were identified in HCT-8^5-FuR^ cells compared to their corresponding untreated control cells. A total of 205 differentially expressed genes (DEGs) were observed in only 5-Fu-resistant cells, and Kyoto Encyclopedia of Genes and Genomes (KEGG) analyses of these genes showed that the “MAPK signaling pathway” was the most enriched pathway (Fig. S3A). Consistently, we found that 5-Fu treatment activated MAPK signaling in two 5-Fu-resistant CRC cells, but not in parental cells, as evidenced by increased phosphorylated MAPK kinase (pMEK) and phosphorylated extracellular signal-regulated kinase (pERK) levels in HCT-8^5-FuR^ and HCT-116^5-FuR^ cells (Fig. S3B). Considering that activated MAPK signaling has been reported to contribute to multiple drug resistance, we hypothesized that 5-Fu triggered MAPK activation is required for the survival of 5-Fu-resistant CRC cells. As expected, the inhibition of MAPK signaling by MEK inhibitor (MEKi) resensitized 5-Fu-resistant CRC cells to 5-Fu (Fig. S3C). Therefore, we investigated whether PE accumulation in 5-Fu-treated resistant CRC cells activates MAPK signaling. As PE has been reported to interact with PEBP1 and shift its binding from RAF1 to IKKα/β [3], we hypothesized that 5-Fu-induced PE accumulation might facilitate the interaction between PEBP1 and IKKα, thereby releasing RAF1 to activate MAPK signaling (Fig. S3D). Our data supported this hypothesis, showing increased PEBP1-IKKα/β association and decreased PEBP1-RAF1 interaction upon 5-Fu treatment (Fig. S3E). These findings suggest that 5-Fu-resistant cells maintain their survival under 5-Fu stimulation by rapidly synthesizing PEs and shifting the affinity of PEBP1 for RAF1 toward IKKa/β, thereby releasing RAF1 to activate MAPK signaling.

We then explored whether emodin could overcome 5-Fu resistance *in vitro* and *in vivo*. Emodin markedly reduced the half maximal inhibitory concentration (IC_50_) of 5-Fu in HCT-8^5-FuR^ and HCT-116^5-FuR^ cells but not in parental cells (Figs. S4A and B), and promoted the anti-proliferative function of 5-Fu in 5-Fu resistance cells but not in their parental cells (Figs. S4C and D). Bliss-score model confirmed the synergy between emodin and 5-Fu in resistant cells (Figs. S4E and F). To confirm the role of emodin in overcoming 5-Fu resistance, we established an HCT-8^5-FuR^ cell-derived xenograft model (Fig. S5A). Unsurprisingly, the combination of emodin and 5-Fu significantly suppressed tumor growth, whereas the monotherapy failed to do so (Figs. 1A, S5B, and S5C). Importantly, no off-tumor toxicity was observed, as evidenced by the absence of histological abnormalities in the major organs, including the heart, liver, spleen, lungs, and kidneys (Fig. 1B). These findings indicated that emodin is a promising alternative for overcoming 5-Fu resistance.

**Fig. 1:**
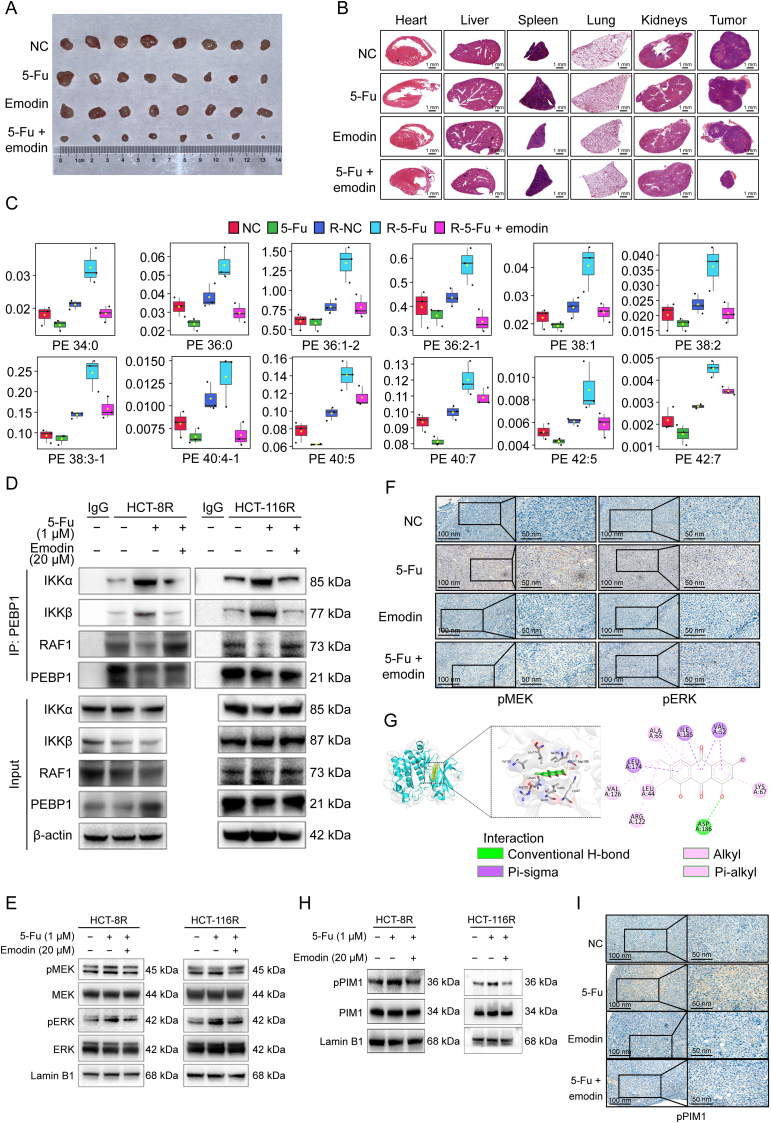
Emodin targets Pim-1 proto-oncogene, serine/threonine kinase (PIM1), suppresses phosphatidylethanolamine (PE), and restores the PE binding protein 1 (PEBP1)-Raf-1 proto-oncogene, serine/threonine kinase (RAF1) interaction to inactivate mitogen-activated protein kinase (MAPK), thereby synergizing with 5-fluorouracil (5-Fu). (A) Image of tumors derived from HCT-8^5-FuR^ xenografted mice with the indicated treatment. (B) Representative hematoxylin-eosin staining in major organs derived from HCT-8^5-FuR^ xenografted mice with the indicated treatment. (C) Box plots representing the fold change of PEs in the indicated groups. (D) Chemoresistance HCT-8 and HCT-116 cells with the indicated treatment were collected and subjected to immunoprecipitation (IP) with anti-PEBP1 antibodies, followed by Western blotting as indicated. (E) Resistant HCT-8 and HCT-116 cells with the indicated treatment were subjected to Western blotting analyses as indicated. (F) Representative immunohistochemistry (IHC) staining of phosphorylated MAPK (pMEK) and phosphorylated extracellular signal-regulated kinase (pERK) in HCT-8^5-FuR^ xenografts with the indicated treatment. (G) Representative image showing the potential binding sites of emodin in PIM1 based on molecular docking analyses. (H) Western blotting analyses of the indicated proteins in 5-Fu-resistant HCT-8 and HCT-116 cells treated with the indicated drug combination. (I) Representative IHC staining of phosphorylated PIM1 in HCT-8 xenografts with the indicated treatment. NC and 5-Fu represent parental HCT-8 cells treated with solvent control or 5-Fu, respectively. R-NC, R-5-Fu, and R-5-Fu-emodin represent 5-Fu-resistant HCT-8 cells treated with solvent control, 5-Fu, or the combination of 5-Fu and emodin. IKKα: inhibitor of nuclear factor kappaB (NF-κB) kinase subunits alpha.

Furthermore, we investigated the molecular mechanism by which emodin overcomes 5-Fu resistance. As expected, emodin induced lipid metabolism remodeling in 5-Fu-treated HCT-8^5-FuR^ cells (Fig. S6A). More importantly, emodin significantly attenuated the majority of PE accumulation caused by 5-Fu in HCT-8^5-FuR^ cells (Fig. S6B), restoring many types of PEs levels close to baseline in untreated HCT-8^5-FuR^ cells (Fig. 1C). Next, we explored whether the attenuating effect of emodin on PE accumulation restores PEBP1-RAF1 interaction. In the presence of emodin, PEBP1 exhibited stronger binding affinity to RAF1 than to IKKα/β in 5-Fu-treated resistant CRC cells (Fig. 1D). Moreover, emodin blocked the activation of 5-Fu-induced MAPK signaling in both HCT-8^5-FuR^ and HCT-116^5-FuR^ cells (Fig. 1E) and in HCT-8^5-FuR^ cell-derived xenograft model (Fig. 1F). To identify the direct target mediating the regulation of PE metabolism by emodin, we utilized SwissTarget and identified five candidates (Table S1), with PIM1 being the only candidate known to regulate the lipid metabolism. Furthermore, the direct association between emodin and PIM1 was confirmed using a cellular thermal shift assay (Fig. S7A). Therefore, we investigated whether PIM1 regulates the chemoresistant phenotype in CRC cells. Notably, co-treatment with the PIM1 inhibitor SMI-4a and 5-Fu phenocopied combination treatment with emodin and 5-Fu (Figs. S7B and C), suggesting that PIM1 may be a crucial direct target. We then performed molecular docking and found that the potential binding site of emodin in PIM1 was extremely close to that of CX-4945 (Fig. 1G), an adenosine triphosphate (ATP)-competitive inhibitor that binds to the ATP binding pocket of PIM1 [4], suggesting that emodin inactivates PIM1 via competitively binding with ATP. Consistently, the levels of phosphorylated PIM1 (pPIM1) were drastically elevated in both HCT-8^5-FuR^ and HCT-116^5-FuR^ cells upon 5-Fu treatment, which were diminished by emodin (Fig. 1H). In addition, in HCT-8^5-FuR^ cell-derived xenografts, 5-Fu treatment led to elevated pPIM1 levels, which was countered by emodin (Fig. 1I). Furthermore, to confirm that PIM1 phosphorylation is required for emodin to inhibit 5-Fu-induced activation of MAPK signaling, we established PIM1^WT^ and PIM1^Y309D^ (a phosphorylated mimic mutant)-reconstituting cells by depleting endogenous PIM1 (Fig. S7D). Using this reconstituting system, we found that co-treatment with emodin and 5-Fu suppressed the activation of MAPK signaling in PIM1^WT^-reconstituting cells but not in cells expressing PIM1^Y309D^ (Fig. S7E), supporting the PIM1 dependency.

In summary, our results revealed that the increased accumulation of PEs induced by 5-Fu treatment was capable of switching the binding partners of PEBP1 from RAF1 to IKKα/β, thereby activating MAPK signaling and further maintaining the chemoresistant phenotype. Emodin suppresses PE accumulation and restores the interaction between PEBP1 and RAF1 to inactivate MAPK signaling by binding to PIM1 and inhibiting its activation. Given that emodin contributes to overcoming the immunopermissive microenvironment, our study highlights emodin as a promising and multi-functional therapeutic candidate for combating 5-Fu resistance in CRC.

## CRediT authorship contribution statement

**Yanyan Chen:** Writing – original draft, Investigation, Conceptualization. **Yanchen Liu:** Investigation, Formal analysis, Data curation. **Zhicheng Gong:** Writing – review & editing, Validation, Investigation. **Zhaohui Huang:** Writing – review & editing, Supervision.

## Ethics approval and consent to participate

All animal studies complied with relevant ethical regulations for animal testing and research, and all procedures were approved by the Institutional Animal Care and Use Committee of Jiangnan University, China (Approval No.: 20230830b0401110 [264]).

## Declaration of competing interest

The authors declare that there are no conflicts of interest.
