# Supplementary material for: Emodin is a novel phosphatidylethanolamine anabolism inhibitor that reprograms lipid metabolism to overcome 5-fluorouracil resistance in colorectal cancer
Source: J Pharm Anal. 2025 May 12;15(10):101343. doi: 10.1016/j.jpha.2025.101343 (PMC12593595; doi:10.1016/j.jpha.2025.101343)
Supplement: Multimedia component 1 [file mmc1.docx]

**LIST OF SUPPLEMENTARY DOCUMENTS**

**Supplementary Fig. S1 Establishment and validation of 5-fluorouracil (5-Fu)-resistant colorectal cancer (CRC) cells**

**Supplementary Fig. S2 5-Fluorouracil (5-Fu) reprograms lipid metabolism in 5-Fu-resistant colorectal cancer (CRC) cells, causing phosphatidylethanolamine (PE) accumulation**

**Supplementary Fig. S3 5-Fluorouracil (5-Fu) reprograms lipid metabolism in 5-Fu-resistant colorectal cancer (CRC) cells, activating mitogen-activated protein kinase (MAPK) via switching PE binding protein 1 (PEBP1) binding partners**

**Supplementary Fig. S4 Emodin restores the in vitro sensitivity of 5-fluorouracil (5-Fu)-resistant colorectal cancer (CRC) cells to 5-Fu**

**Supplementary Fig. S5 Emodin sensitizes 5-fluorouracil (5-Fu)-resistant colorectal cancer (CRC) cell-derived xenografts to 5-Fu *in vivo***

**Supplementary Fig. S6 Emodin suppresses phosphatidylethanolamine (PE) accumulation**

**Supplementary Fig. S7 Emodin targets Pim-1 proto-oncogene, serine/threonine kinase (PIM1)**

**Supplementary Table. S1 Potential direct targets of emodin predicted by SwissTarget**

**
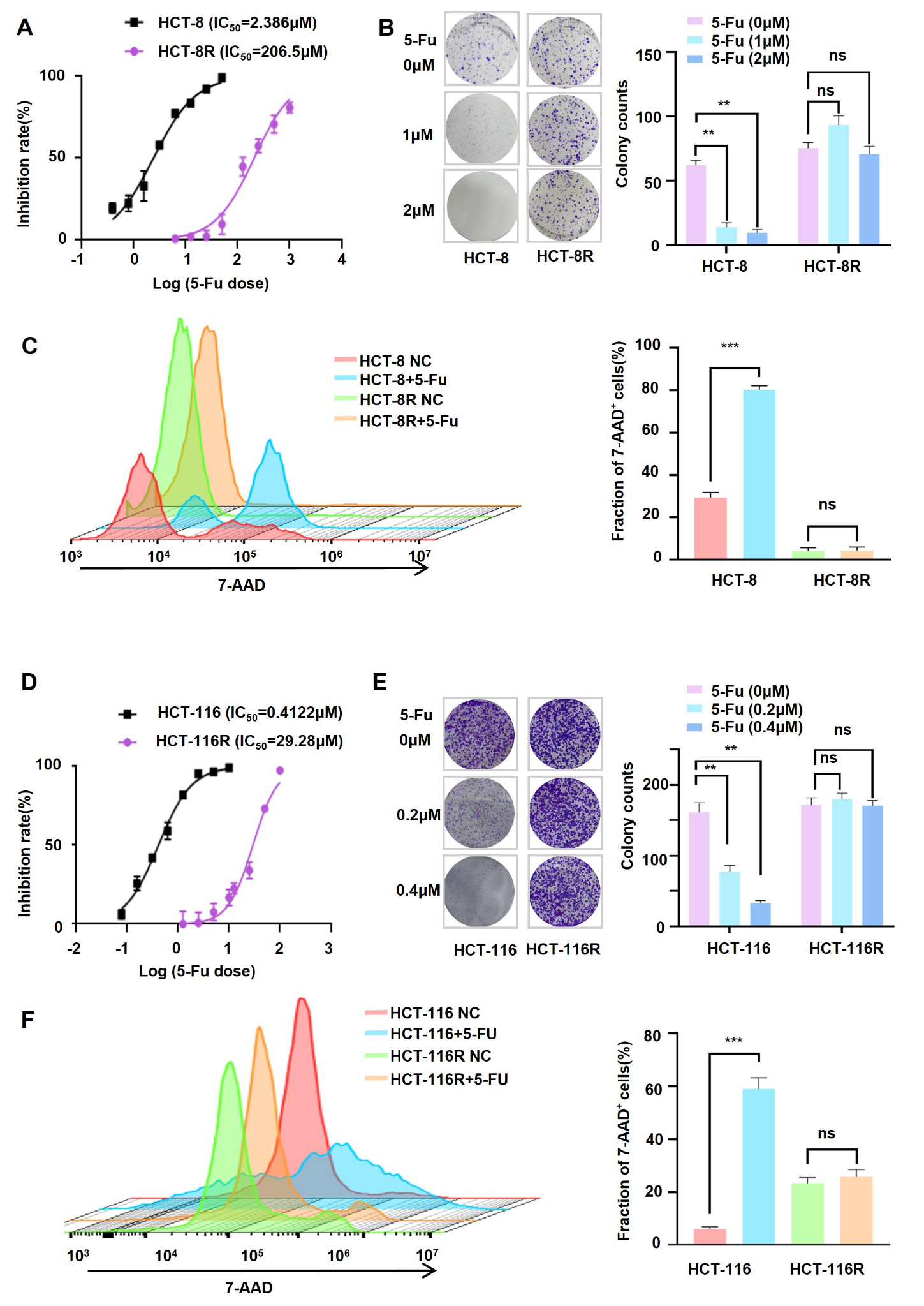
**

**Fig. S1 Establishment and validation of 5-fluorouracil (5-Fu)-resistant colorectal cancer (CRC) cells**

(A) Cell counting kit-8 (CCK-8) analyses of HCT-8 and HCT-8^5-FuR^ cells treated with gradient doses of 5-Fu. (B) Clonogenic assay in the indicated cells with vehicle or 5-Fu treatment. Right panel: Quantification of colony numbers. (C) Apoptosis in HCT-8 and HCT-8^5-FuR^ cells treated with vehicle or 5-Fu was examined by flow cytometry. (D) CCK-8 assay of HCT-116 and HCT-116^5-FuR^ cells with gradient dose of 5-Fu treatment. (E) colony formation assay of HCT-116 and HCT-116^5-FuR^ cells treated with vehicle and 5-Fu. (F) Apoptosis in HCT-116 and HCT-116^5-FuR^ cells with the indicated treatments were measured by flow cytometry. *n* = 3. Data are presented as means ± standard deviation. *** *P* < 0.001, ** *P* < 0.005, ns = no significance. NC: negative control; 7-AAD: 7-aminoactinomycin D.


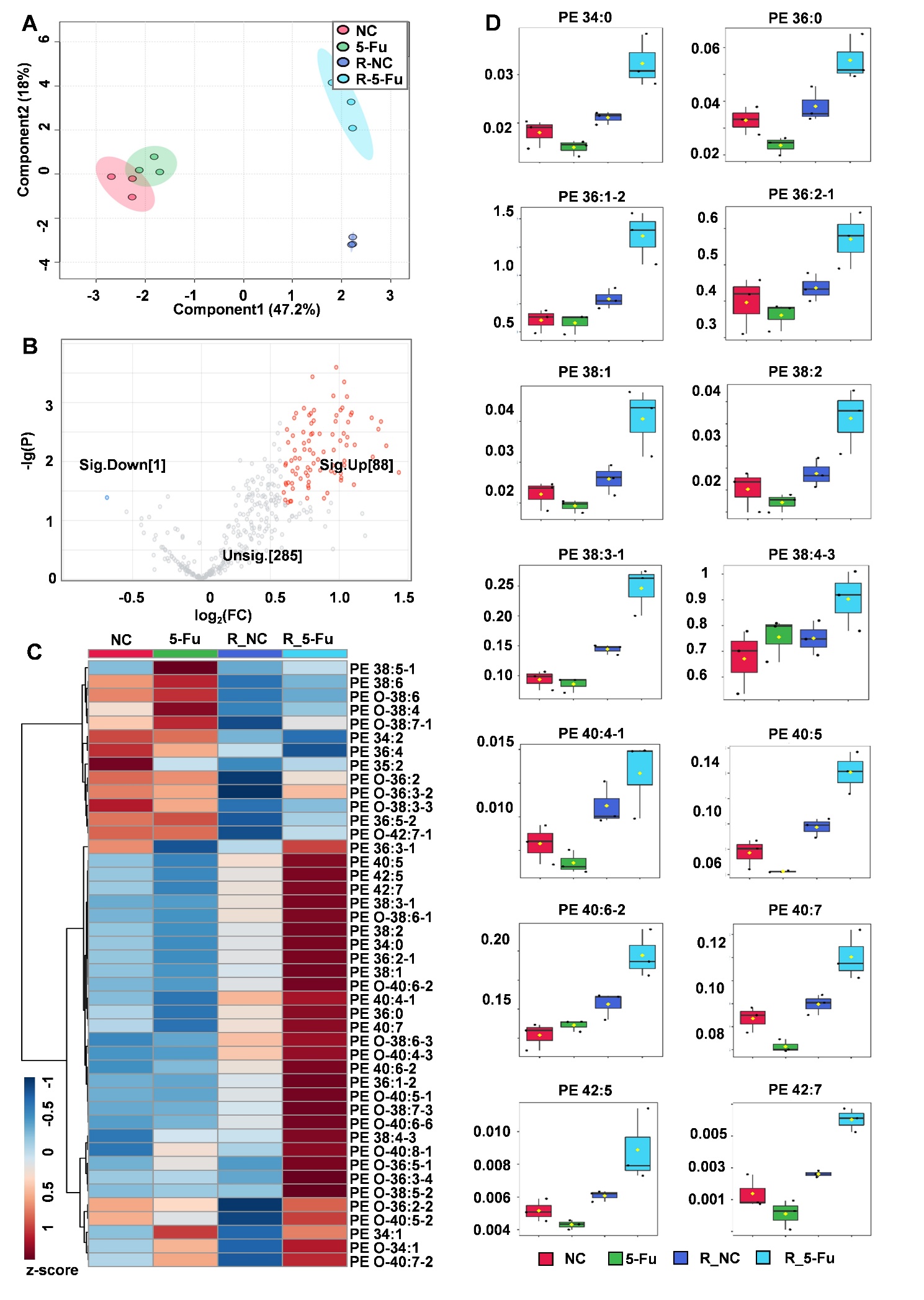


**Supplementary Fig. S2 5-Fluorouracil (5-Fu) reprograms lipid metabolism in 5-Fu-resistant colorectal cancer (CRC) cells, causing phosphatidylethanolamine (PE) accumulation**

(A) Scores plot illustrating the overall alterations of lipids in parental and 5-Fu-resistant HCT-8 cells with acute 5-Fu treatment based on principal components analysis. (B) Volcano plot showing the detected lipid components in 5-Fu-resistant HCT-8 cells upon acute 5-Fu treatment. (C) Heatmap presenting the fold change of indicated PEs in parental or 5-Fu-resistant HCT-8 cells treated with vehicle or 5-Fu treatment. (D) Box plots representing the fold change of phosphatidylethanolamines (PEs) in the indicated groups. NC and 5-Fu represent parental HCT-8 cells treated with solvent control or 5-Fu, respectively. R-NC and R-5-Fu represent 5-Fu-resistant HCT-8 cells treated with solvent control or 5-Fu, respectively. FC: fold change.


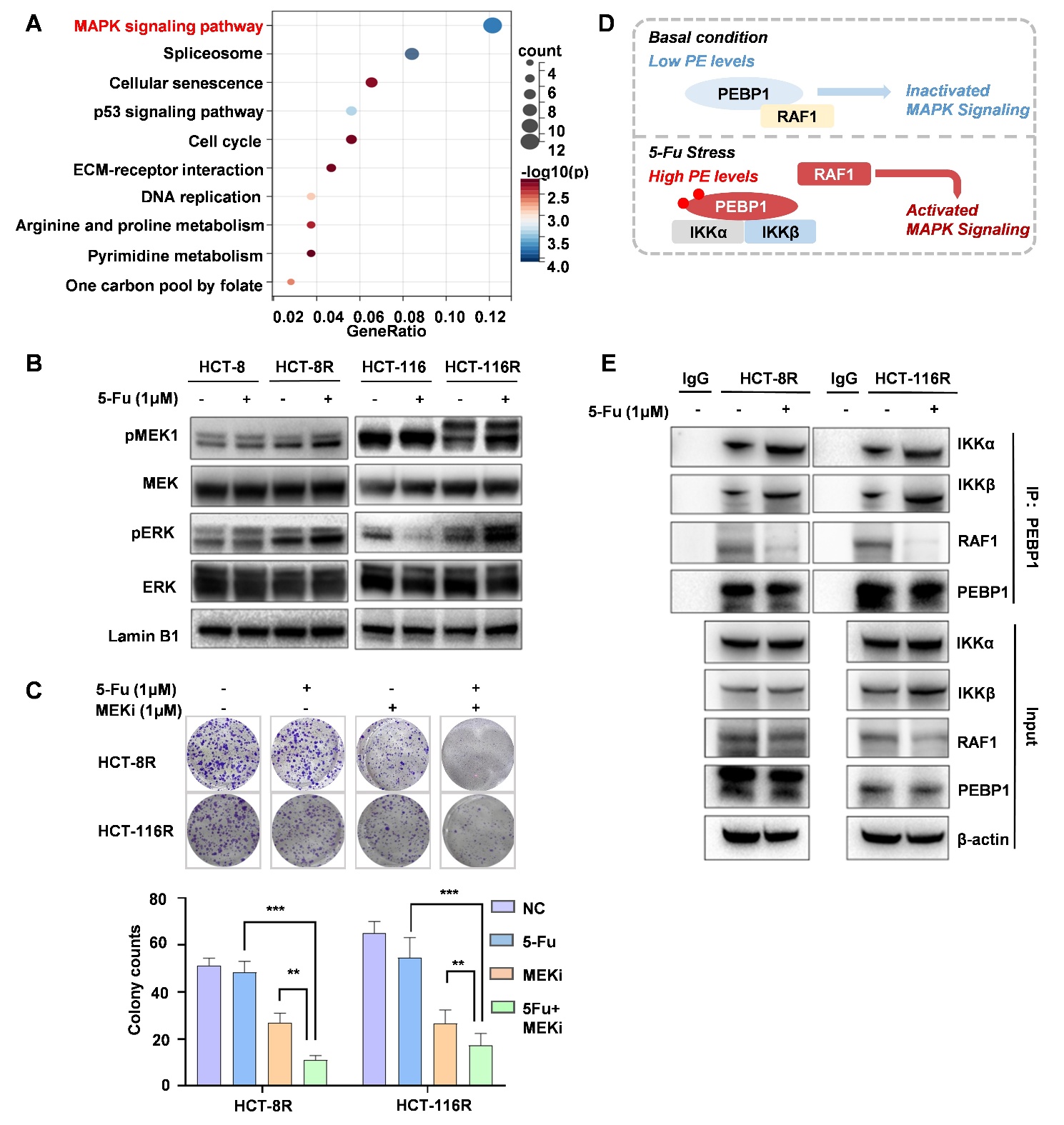


**Supplementary Fig. S3 5-Fluorouracil (5-Fu) reprograms lipid metabolism in 5-Fu-resistant colorectal cancer (CRC) cells, activating mitogen-activated protein kinase (MAPK) via switching PE binding protein 1 (PEBP1) binding partners**

(A) Kyoto Encyclopedia of Genes and Genomes (KEGG) analyses of chemoresistance-specific differentially expressed genes (DEGs) induced by 5-Fu treatment. (B) Western blotting analysis of indicated proteins in parental and 5-Fu-resistant HCT-8 and HCT-116 cells treated with vehicle or 5-Fu. (C) Clonogenic assay in parental and 5-Fu-resistant HCT-8 and HCT-116 cells with indicated treatment. (D) Proposed molecular mechanism of 5-Fu resistant phenotype attributed by accumulated PEs. (E) Immunoprecipitation with anti-PEBP1 antibodies followed by Western blotting analyses of lysates derived from parental and 5-Fu-resistant CRC cells treated with vehicle or 5-Fu. NC: negative control; ECM: extracellular matrix; pMEK: phosphorylated mitogen-activated protein kinase kinase 7; pERK: phosphorylated extracellular signal-regulated kinase; MEKi: mitogen-activated protein kinase kinase inhibitor; RAF1: Raf-1 proto-oncogene, serine/threonine kinase; IKKα: inhibitor of nuclear factor kappa-b kinase subunits alpha; IP: immunoprecipitation.


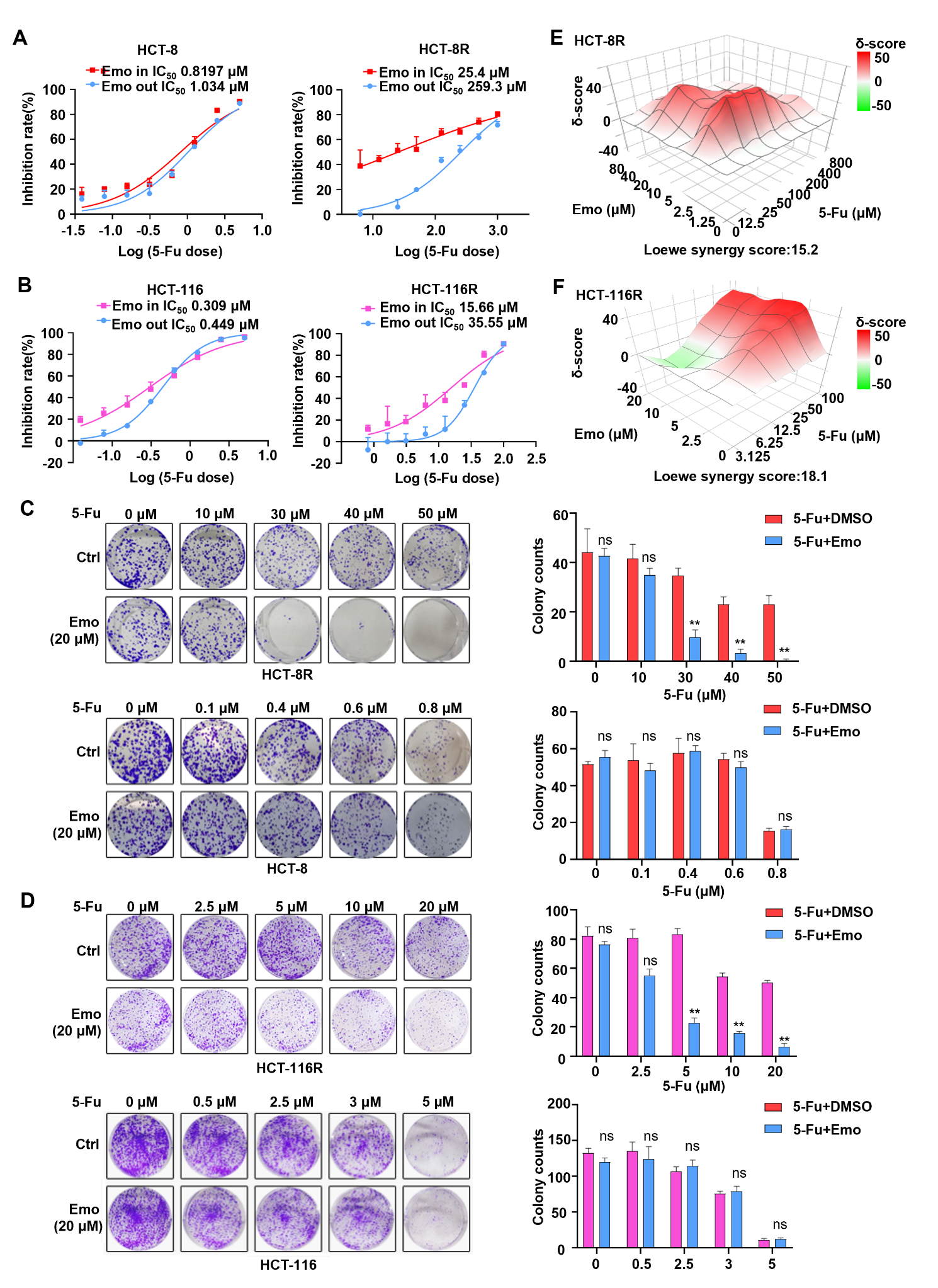


**Fig. S4 Emodin restores the in vitro sensitivity of 5-fluorouracil (5-Fu)-resistant colorectal cancer (CRC) cells to 5-Fu**

(A-B) Half maximal inhibitory concentration (IC_50_) determination in parental and 5-Fu-resistant HCT-8 (A) and HCT-116 (B) cells in the presence of DMSO or Emodin. (C-D) Clonogenic assays in parental and 5-Fu-resistant HCT-8 (C) and HCT-116 (D) cells with the indicated treatment. (E-F) Chemoesistant HCT-8 (E) and HCT-116 (F) cells with indicated treatment were subjected to cell counting kit-8 analyses. Synergetic scores were calculated using Bliss-Score model. *n* = 3. Data are presented as means ± standard deviation. ** *P* < 0.005, ns = no significance. DMSO: dimethyl sulfoxide; Emo: emodin.


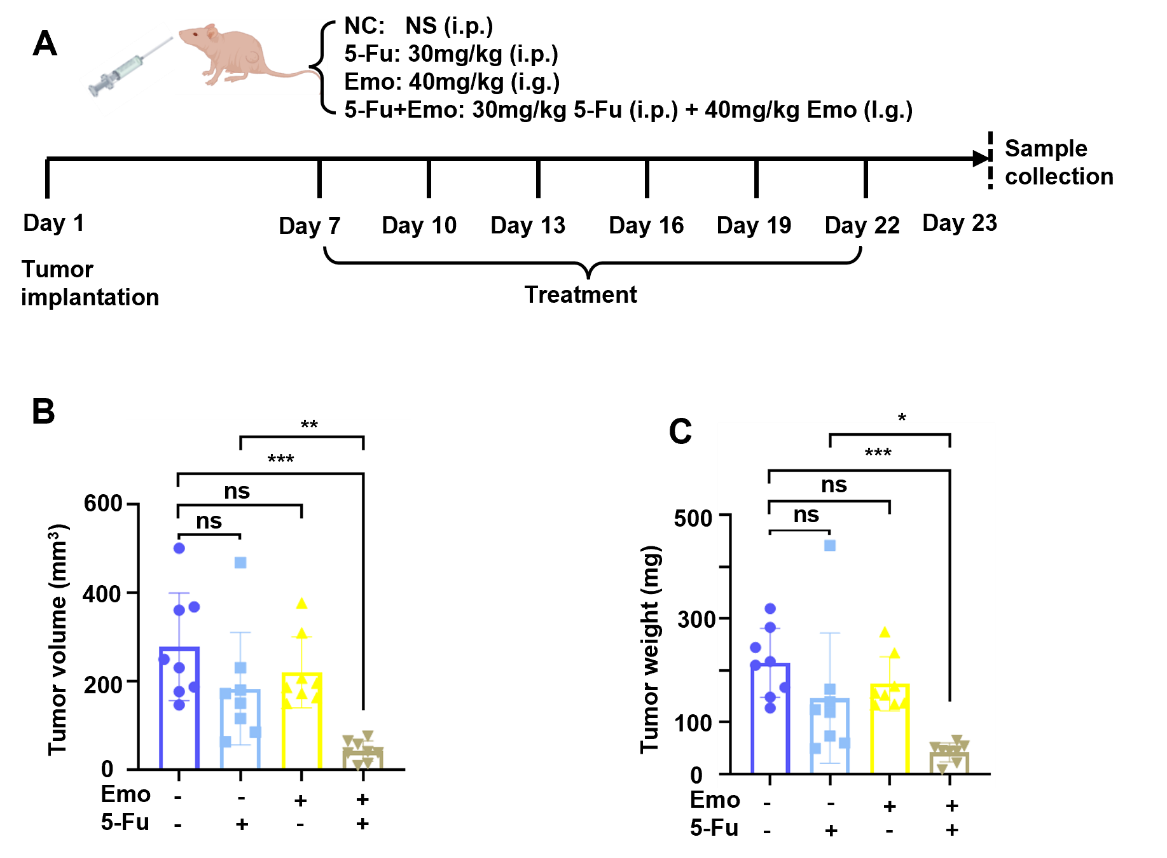


**Fig. S5 Emodin sensitizes 5-fluorouracil (5-Fu)-resistant colorectal cancer (CRC) cell-derived xenografts to 5-Fu *in vivo***

(A) Schematic diagram of the *in vivo* experiment. (B) Quantification of tumor volume. (C) Quantification of tumor weight (D). *n* = 8. Data are presented as means ± standard deviation. *** *P* < 0.001, ** *P* < 0.005, * *P* < 0.05, ns = no significance. NC: negative control; NS: normal saline; i.p.: intraperitoneal; i.g.: intragastric; Emo: emodin.


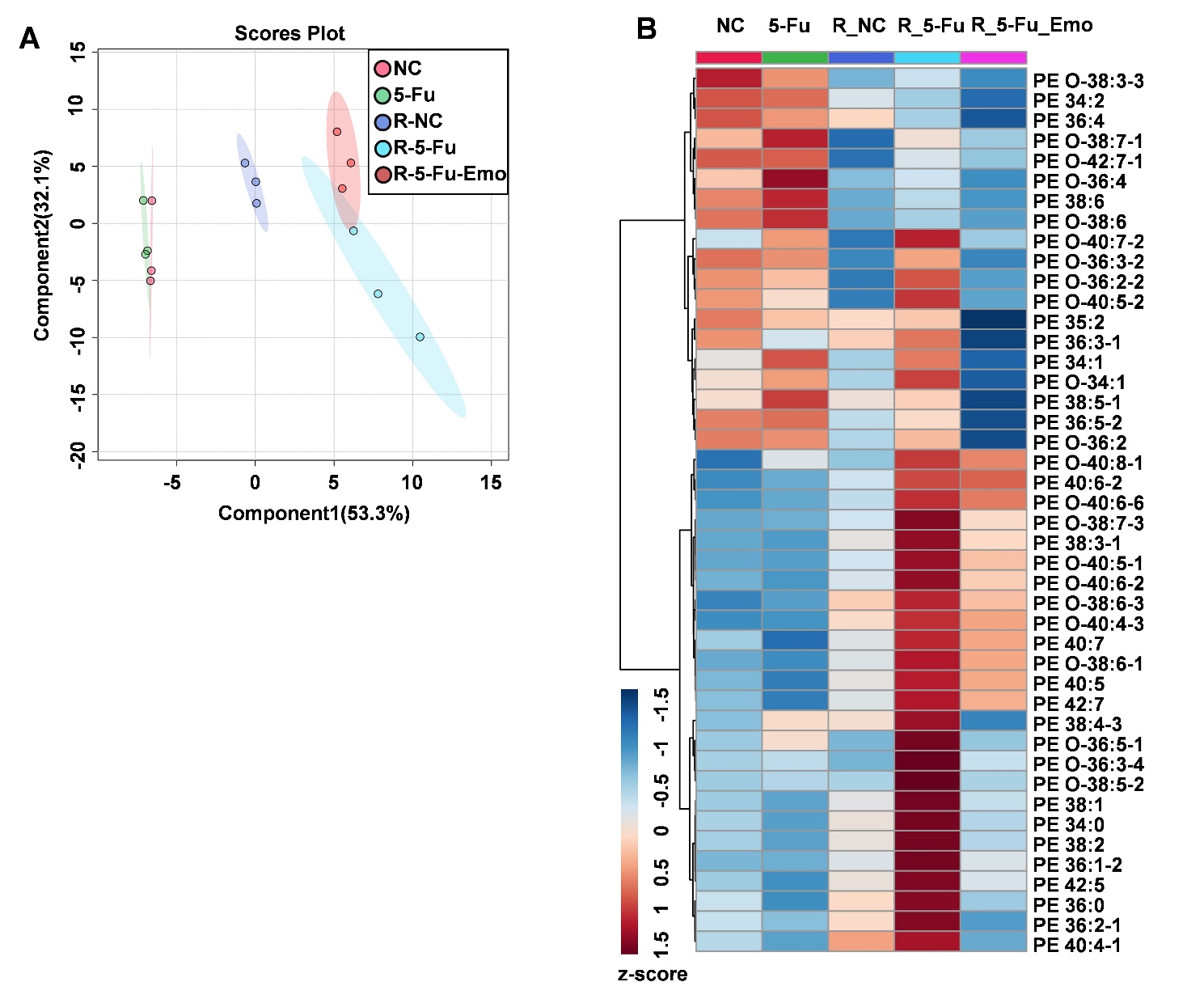


**Fig. S6 Emodin suppresses phosphatidylethanolamine (PE) accumulation**

(A) Scores plot displaying the overall alterations of lipids in parental and 5-fluorouracil (5-Fu)-resistant HCT-8 cells with the indicated treatment based on principal components analysis. (B) Heatmap summarizing the fold change of indicated phosphatidylethanolamines (PEs) in parental and 5-Fu-resistant HCT-8 cell with the indicated treatment. NC and 5-Fu represent parental HCT-8 cells treated with solvent control or 5-Fu, respectively. R-NC, R-5-Fu and R-5-Fu-Emo represent 5-Fu-resistant HCT-8 cells treated with solvent control, 5-Fu, or the combination of 5-Fu and emodin.


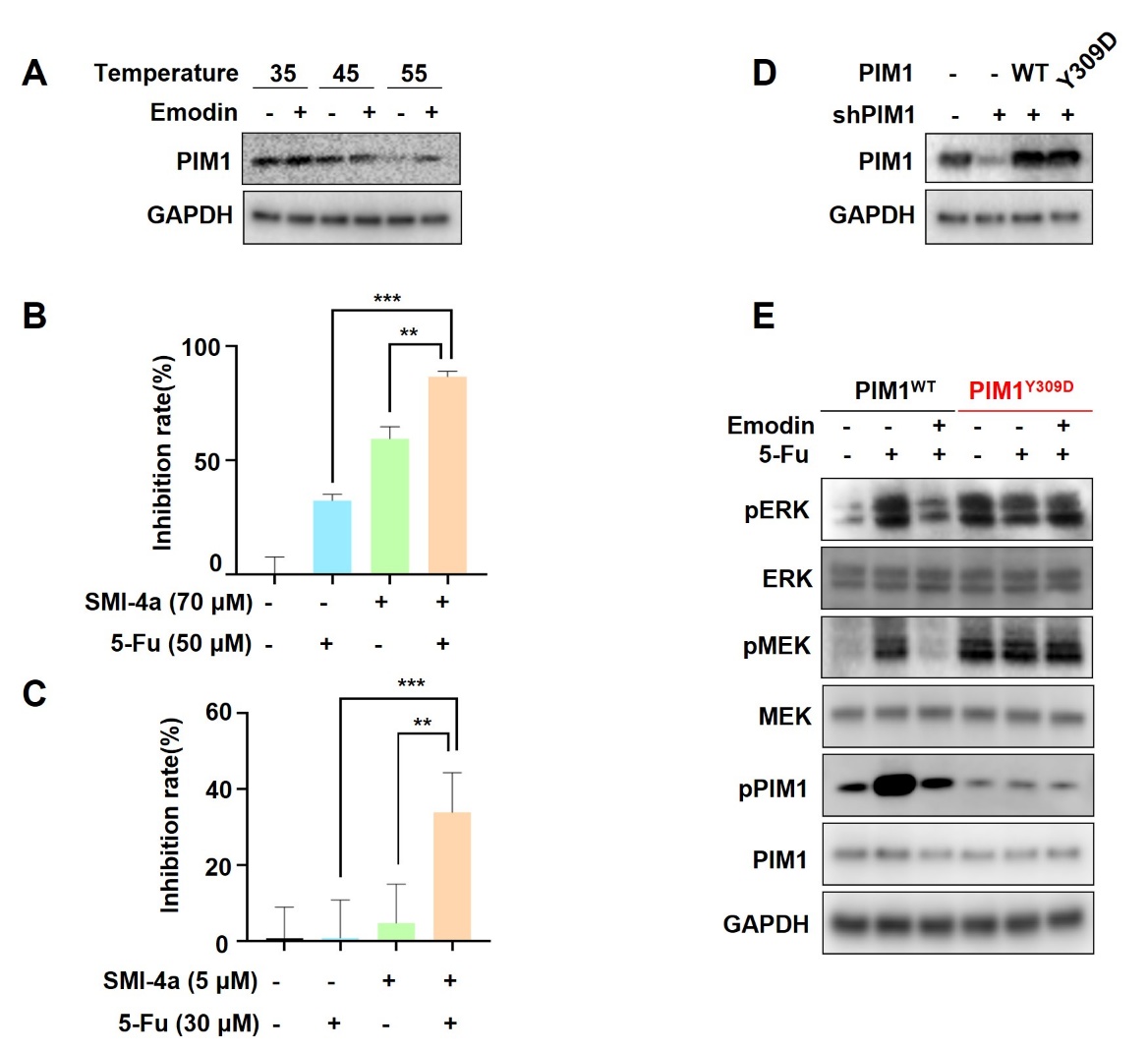


**Fig. S7 Emodin targets** **Pim-1 proto-oncogene, serine/threonine kinase (PIM1)**

(A) HCT-8^5-FuR^ cells treated with emodin were heated at indicated temperature, followed by Western blotting analyses. (B-C) Cell counting kit-8 analyses of HCT-8^5-FuR^ (B) and HCT-116^5-FuR^ (C) cells with the indicated treatment. (D) HCT-8^5-FuR^ cells were infected with lentivirus encoding indicated constructs, followed by Western blotting analysis as indicated. (E) Wild-type PIM1 and PIM1^Y309D^ reconstituting HCT-8^5-FuR^ cells were treated with emodin (20 µM) and 5-Fu (1 µM), followed by Western blotting analyses as indicated. *** *P* < 0.001, ** *P* < 0.005. WT: wild type; pERK: phosphorylated extracellular signal-regulated kinase; pMEK: phosphorylated mitogen-activated protein kinase kinase 7; pPIM1: phosphorylated Pim-1 proto-oncogene, serine/threonine kinase.

**Table S1 Potential direct targets of emodin predicted by SwissTarget**

| Target | Common name | Uniprot ID | ChEMBL ID | Target Class | Probability* | Known actives (3D/2D) |
| --- | --- | --- | --- | --- | --- | --- |
| Estrogen receptor alpha | ESR1 | P03372 | CHEMBL206 | Nuclear receptor | 1 | 71 / 19 |
| Serine/threonine-protein kinase PIM1 | PIM1 | P11309 | CHEMBL2147 | Kinase | 1 | 5 / 1 |
| Estrogen receptor beta | ESR2 | Q92731 | CHEMBL242 | Nuclear receptor | 1 | 87 / 17 |
| Casein kinase II alpha | CSNK2A1 | P68400 | CHEMBL3629 | Kinase | 1 | 5 / 3 |
| Protein-tyrosine phosphatase 4A3 | PTP4A3 | O75365 | CHEMBL4162 | Phosphatase | 1 | 1 / 1 |
